# *Eucommia* polysaccharides alleviate experimental colitis by reshaping colonic microbiota composition, metabolites, and modulating the IL-17 signaling pathway

**DOI:** 10.3389/fmicb.2026.1769429

**Published:** 2026-03-25

**Authors:** Wenna Yao, Shuo Yan, Ruilin Du, Yulong Zhao, Huimin Zhang, Bincheng Wang, Xinyue Cheng, Zimeng Ma, Siqin Bao, Xihe Li, Yongli Song

**Affiliations:** 1 Research Center for Animal Genetic Resources of Mongolia Plateau, College of Life Sciences, Inner Mongolia University, Hohhot, China; 2 The State Key Laboratory of Reproductive Regulation and Breeding of Grassland Livestock, College of Life Sciences, Inner Mongolia University, Hohhot, China

**Keywords:** East Friesian sheep fecal microbiota, *Eucommia* polysaccharides, FMT, metabolites, microbiota, mouse colitis model

## Abstract

Intake of plant polysaccharides are associated with a reduced risk of ulcerative colitis (UC). *Eucommia* polysaccharides (EUPs) are promising nutritional supplements with notable antibacterial, anticancer, and anti-inflammatory properties. They exhibit a moderate molecular weight and are resistant to gastric acid degradation. Yet, the action mechanisms of microorganisms and metabolites in EUPs for UC treatment remain incompletely elucidated. The current study was formulated to assess the therapeutic efficacy of EUPs against fecal microbiota transplantation (FMT)-induced colitis, focusing on comparing the effects of low-dose and high-dose EUPs. After East Frisian sheep received dextran sulfate sodium (DSS) intervention, their fecal microbiota was used to prepare fecal bacterial suspensions for mouse FMT. Administration of high-dose EUPs alleviated key colitis symptoms, improved colonic epithelial barrier, preserved microbial diversity, and significantly reduced harmful bacterial (e.g., *g_Klebsiella and g_unidentified_Enterobacteriaceae*). Furthermore, this treatment significantly enriched the bile secretion pathways, particularly those involving deoxycholic acid (DCA) and hyodeoxycholic acid (HDCA). Additionally, DCA and HDCA were significantly negatively correlated with *g_unidentified_Enterobacteriaceae* and *g_Klebsiella*, and these two bile acids were also negatively correlated with key genes associated with the IL-17 signaling pathway. Overall, this study elucidates that EUPs ameliorate FMT-induced colitis in a mouse model via restoring gut microbes and metabolites and modulating the IL-17 pathway, thereby providing novel insights into therapeutic strategies for UC.

## 1 Introduction

IBD is mainly divided into two subtypes (ulcerative colitis and Crohn’s disease), with ulcerative colitis (UC) representing the more prevalent subtype (Xia et al., 2024). In recent decades, the incidence and prevalence of UC have significantly increased worldwide (Li et al., 2021). Enteritis is difficult to cure and can cause chronic diseases in animals, which has a substantial negative influence on human health and livestock agricultural output (Yan et al., 2024). As a consequence, it has long emerged as a focus in investigations into human health and metabolic balance (Gentile and Weir, 2018). DSS-elicited colitis in lab animals is a chemically induced model that recapitulates the human UC-associated pathological phenotype (Fu et al., 2022). Specifically, the gastrointestinal tract is inhabited by trillions of different types of pathogenic, mutualistic, and commensal microbes that form the gut microbiota, a complex ecosystem critical for maintaining host physiological balance and regulating the pathogenic mechanisms of gut inflammatory disorders conditions (Wrzosek et al., 2021). The increase in inflammation caused by intestinal microbiota can damage the intestinal barrier (Huang et al., 2021). On the contrary, breakdown of the gut epithelial barrier drives disease occurrence and progression by permitting the buildup of gut-resident microbiota-associated molecular patterns (Carloni et al., 2021). Fecal microbiota transplantation from DSS-induced sheep recapitulates the pathological phenotypes associated with intestinal inflammatory conditions, highlighting the pathogenic role of dysregulated ovine gut microbiota (Li and Grobelna, 2019). FMT recipient mice manifested typical colitis symptoms, such as extensive inflammatory cell infiltration into the colonic mucosa and reduced intestinal mucus production, with these pathological manifestations mirroring those of mice with DSS-induced colitis (Zhang et al., 2025). Hence, further in-depth studies on the etiological agents of DSS-elicited enteritis and host defensive mechanisms are necessary to formulate efficient approaches for its treatment and prevention.

UC is characterized by an exceptionally complex pathogenesis involving the interplay of multiple etiological factors. Moreover, against the backdrop of UC pathogenesis being tightly linked to gut microbiota dysregulation, a lot of pharmacological, dietary, and microbiological methods have been employed to regulate the interactions among the gut microbiota, including metabolic changes, adjustments to humoral regulatory systems, to the translocation of immune-related factors (Zhao et al., 2022a, 2022b; Zhao X. et al., 2022). For instance, the gut microbiota decomposes dietary tryptophan into indole derivatives to trigger aryl hydrocarbon receptor activation, ultimately ameliorating alcohol-induced intestinal injuries (Wrzosek et al., 2021). Intestinal inflammation induced the disruption of gut microecological homeostasis, compromises intestinal submucosa integrity, and elevates the abundance of pro-inflammatory mediators within the intestinal microenvironment, including cytokines, as well as pathogen-associated molecular patterns lipopolysaccharide (Abbasi et al., 2019). The symbiotic bacteria and microbial products enter the intestinal wall, activating immune cells and producing cytokines (interleukin-17 and tumor necrosis factor-α) (Le Berre et al., 2023). Patients with UC also exhibit gut microbiota dysbiosis, characterized by decreased and elevated ratios of Bacteroidetes to Bacillota, as well as reduced microbiota diversity (Kong et al., 2019). Systemic inflammation induces memory impairment via diminishing the IL-17 signaling pathway (Zhang et al., 2014). Interleukin-17 is acknowledged as a pivotal mediator driving the pathophysiology in experimental colitis (Serrano et al., 2019). IL-6, on the other hand, exerts most of its pro-inflammatory functions via trans-signaling, leading to downstream activation of the JAK-STAT3 pathway during colonic inflammation (Serrano et al., 2019). JUN and FOS are also key components of the IL-17-mediated cascade to mediate the transcription of inflammation-related target genes (Schraml et al., 2009). The widespread application of the interplay analysis among microorganisms, metabolites, and the host also offers a novel perspective for deciphering the mechanism through which dietary interventions ameliorate UC.

The drugs used to treat UC mainly include corticosteroids, immunomodulators, and biologics (Jing et al., 2021). Moreover, traditional Chinese medicine (TCM), including *Curcumin*, *Tripterygium wilfordii*, *Andrographis paniculata*, and *Cannabinoids*, has shown efficacy in intestinal inflammation (Chen et al., 2021). However, most of these agents are non-targeted and can cause serious adverse effects (Herfarth and Vavricka, 2022). Accordingly, safer dietary supplements and functional foods are required. For instance, prebiotics (e.g., *fructooligosaccharides* and *galactooligosaccharides*) are mostly small-molecule oligosaccharides with simple structures, strongly target beneficial bacteria proliferating with low cost and efficiency (Keshteli et al., 2019). Safer polysaccharides in dietary supplements and functional foods are crucial to both colitis prevention and the design of novel, efficient, and safe intervention regimens. Studies have demonstrated that *pectin* polysaccharides can facilitate cytokine secretion and boost immune regulation-related signaling pathways (Guo et al., 2025). By serving as potential prebiotics, the active fractions of *Astragalus* polysaccharides exert anti-colitis effects while enhancing immune function (Huo et al., 2024). Gut microbiota and mucus O-glycans restoration by *Lycium barbarum arabinogalactan* contributes to the alleviation of intestinal mucosal damage in mice (Zhao T. et al., 2024; Zhao Y. et al., 2024). *Eucommia* polysaccharides, as a natural plant-derived prebiotic derived from the plant *Eucommia ulmoides* Oliv, not only exert prebiotic functions but also possess anti-inflammatory and antioxidant bioactivities, which further contribute to targeted regulation of gut microbiota and improved immune function (Feng et al., 2016). *Eucommia ulmoides* Oliv (EU), a medicinal plant with a history of over 2000 years, yields polysaccharides that serve as the core active components in its active ingredients. Purification of *Eucommia ulmoides* polysaccharides via the hot water extraction method, Sevage method, and Sephadex G-200 gel column elution yields products with 89.12% total sugar, and the plant also contains abundant active components (e.g., iridoids, polyphenolic acids) that are more concentrated than those in most common medicinal plants (Song et al., 2023). At the same time, these constituents exhibit antibacterial, antioxidant, and anti-inflammatory properties (Ye et al., 2023). EUPs are mainly composed of arabinose (26.61%), galacturonic acid (25.1%), galactose (19.35%), rhamnose (16.13%), and glucose (12.9%), with arabinose accounting for a prominent proportion significantly higher than that in other plant polysaccharides (Cui et al., 2023). EUPs can prevent memory impairments triggered by systemic inflammation through the regulation of the inflammation pathway (Gao et al., 2020). Interestingly, it is reported that EUPs can reduce the damage by mediating the regulation of immune responses (Ye et al., 2023). However, the ecological mechanism and specific molecular pathways through which *Eucommia* polysaccharides modulate alterations in intestinal microbiota composition, bile acid metabolism, and are correlated to signaling pathways in fecal microbiota transplantation-induced colitis remain largely undetermined.

Here, we sequenced the colonic microbiome, metabolites, and host gene expression to assess the therapeutic and preventive efficacy of EUPs in FMT-induced murine colitis. Our data show administration of EUPs can ease colitis by improving the intestinal barrier integrity, and further alter the colonization potential of intestinal probiotic bacteria, maintain intestinal microbial balance, and in turn raise the production of gut metabolites (such as bile acids). Furthermore, EUPs alleviate FMT-induced colitis in mice, with this protective effect mechanistically correlated with the modulation of the intestinal microbial populations-metabolic axis. Our key findings highlight the intestinal microbiota-metabolic axis as a novel treatment target for UC and validate EUPs as a promising, multifaceted candidate for treating this disorder.

## 2 Materials and methods

### 2.1 Drugs and chemical reagents

For the current experimental study, *Eucommia* polysaccharides (purity >98%, S27810) was purchased from Shanghai Yuanye Bio-Technology Co., Ltd. (Shanghai, China). *Eucommia* polysaccharides (EUPs) were obtained from a commercial source, and the same batch was used for all experiments to ensure structural consistency and experimental reproducibility. DSS (MP Biomedicals, 160110, 36–50 kDa, Illkirch, France), Qualitative detection kit for fecal occult blood (O-toluidine method, Solarbio, BC8270, Beijing, China), Anti-β-ACTIN (Yeasen, 30102ES40), Anti-ZO-1 (Proteintech, 21773-1-AP), Secondary antibodies acquired from YESEN Biotech Co., Ltd. (3010ES40/34101ES60).

### 2.2 Study experimental design and technical approaches

#### 2.2.1 Study design of animal experiments

Fecal samples were collected from East Friesian sheep with colitis, which were induced by dextran sulfate sodium according to our group’s previously established protocol (Yan et al., 2024), and all these samples were preserved in our laboratory. Specifically, colitis was induced in sheep by DSS administration for 5 days as previously established, and the colitis phenotype of donor sheep was rigorously validated through a combination of clinical sign assessment and histopathological characterization in Yan et al. (2024) published in microbiome. Specific-pathogen-free male C57BL/6JNifdc mice, weighing 20 ± 3 g and aged 6–8 weeks, were employed in this research (Weitong Lihua Experimental Animal Technology, Beijing, China). The mouse rearing environment’s temperature, light cycle, dietary supply, humidity parameters, and antibiotic treatment procedures can also be directly adjusted based on the methods described before (Yan et al., 2024). Subsequently, all four groups of mice were treated with antibiotics and randomly separated into four experimental cohorts (*n* = 10/group) and underwent interventions as follows for this designated duration: (1) Control group (PBS): Mice were administered with 200 μL PBS for 10 consecutive days; (2) The ED_PBS group (FMT alone): Mice were given oral gavage of 200 μL fecal microbiota suspension (1 g feces/5 mL sterile PBS) once daily for 7 days, along with concurrent daily oral administration of 200 μL PBS for 10 consecutive days; (3) The ED_LEUP group (FMT + low-dose EUPs): mice were given the same dose of fecal microbiota suspension daily for 7 days, and simultaneously received daily oral gavage of 250 mg/kg body weight EUPs for 10 days; (4) The ED_HEUP group (FMT + high-dose EUPs): Mice were administered the same dose of fecal liquid for 7 days, with simultaneous daily oral gavage of 500 mg/kg body weight EUPs for 10 days. Notably, the Control group was subjected to the same oral gavage route as the FMT and EUPs treatment groups to ensure consistency in administration methods across all groups. Pre-experiments demonstrated that 250 mg/kg EUPs could moderately enhance mice’s dietary intake, body weight, and DAI, with the 500 mg/kg dose achieving the best outcomes. The dosages of EUPs were determined based on relevant prior studies and our preliminary experimental data (Gao et al., 2020). During the entire experimental duration, the mice were assessed daily for alterations in body condition and weight, water intake, food intake, perianal soiling, fecal bleeding, and stool consistency (9:00 to 10:00) (Yan et al., 2024). After anesthetizing the mice with isoflurane, blood was collected from the posterior orbital vein (in compliance with the Institutional Animal Care and Use Committee guidelines), followed by 1 h of room-temperature incubation and centrifugation (4,000 rpm, 15 min) and 4 °C for serum preparation. After weighing the spleen, part of the distal colon tissue was fixed, while all the remaining fresh samples (colon tissue specimens, feces, and colonic contents) were frozen at −80 °C for subsequent assays. Fresh sheep or mouse samples must be processed within 30 min (Anaerobes). The key is to maintain the original microbial state to prevent environmental factors (such as pH value fluctuations, water loss, and oxygen exposure) from causing changes in the community structure and function of the samples, ensuring the authenticity, comparability, and repeatability of subsequent experiments.

#### 2.2.2 Conditions and ethics for animal experiments

All animal-based experiment procedures performed in this research complied with the National Research Council’s Guide (Care and Use of Laboratory Animals). These protocols were additionally approved by the Institutional Animal Care and Use Committee of Inner Mongolia University, located in China, bearing the approval identifier 2022-0003 (NMGDX (Wu)) and the official approval date on December 3, 2022.

### 2.3 Preparation of fecal microbiota solution from donors

Fecal microbial suspensions, with slight modifications, were based on previously published protocols (Yan et al., 2024). Briefly, randomly collect feces from four sheep, weigh 2.5 g accurately per sheep to get a 10 g combined sample, and homogenize with 50 mL sterile PBS (1 g feces/5 mL). Subsequently, stir with a magnetic stirrer (4 °C) for 20 min (1,000 r/min) to achieve thorough homogenization, yielding a particle-free suspension with a uniform emulsified texture. The mixture is then filtered through double-layered sterile gauze to remove undigested debris. After filtration, the filtrate was subjected to centrifugation (2,000 × g, 5 min), with the recovered bacterial pellet was resuspended in an equal sterile PBS (equal volume) to obtain a uniformly standardized fecal bacterial suspension for subsequent transplantation. Serial dilution was used for bacterial concentration calibration, and a turbidimetric method (purchased from Hunan Bikerman Holdings Co., Ltd.) was applied to measure fecal bacterial counts (1 × 10^8^ CFU/mL). For long-term storage, sterile glycerol was added to the suspension at a final volume ratio of 1:10 (glycerol: fecal bacterial suspension). Until subsequent use, the mixture was aliquoted and stored at −80 °C.

### 2.4 Organ index

Spleen and colon tissues were excised, and their organ indices were computed using the specified formula (Xie et al., 2022).

### 2.5 Disease activity index (DAI) score

Mice’s DAI and body weight were quantified and documented for each mouse across all experimental groups. As defined in Table 1, the final DAI value is evaluated as the average of these three distinct parameters (Xie et al., 2022).

**Table 1:** DAI score.

Score	Weight loss (%)	Stool consistency	Blood in stool
0	0	Normal	Normal
1	1–5	Soft	Normal
2	5–10	Fluid	Occult blood
3	10–20	Fluid	Occult blood
4	≥20	Diarrhea	Gross bleeding

### 2.6 H&E staining

For histopathological assessment, five distal colon tissue samples in each group were first fixed in 4% paraformaldehyde solution, followed by paraffin wax embedding and sectioning into 5 μm-thick slices, and subjected to staining based on experimental requirements (Solarbio, G1120). Every detailed staining procedure was rigorously performed in line with the reagent kit protocols provided.

### 2.7 Histological analysis score (HAI) of colon

Each sample was subjected to H&E staining, followed by histological scoring of the stained sections. Four visual fields per slide were chosen for histopathological scoring, with all pathological assessments performed by a pathologist to guarantee the objectivity of the results. A microscope operating at 200× magnification was used to capture the images.

### 2.8 AB-PAS staining procedure

Staining via the AB-PAS reagent was performed to visualize mucin in the intestinal epithelium (Solarbio, G1285), and the specific staining steps followed those reported in our previous research (Yan et al., 2024).

### 2.9 Immunohistochemistry-based (IHC) staining procedure

5 μm-thick distal colon tissue sections (each group incorporates three experimental replicates and three technical replicates to ensure reliability) were heated at 60 °C, dewaxed, and processed following the H&E staining. Sodium citrate buffer was used for pH 6.0 antigen retrieval (100 °C, 20 min) (Yeasen, 36311ES50). Once cooled, the sections were washed with pH 7.4 PBS (5 min, 3 times). Then, sections were first exposed to 3% H₂O₂ for 15 min, then permeated with 0.1% Triton X-100 diluted in PBS (15 min), and finally washed using PBS (5 min, 3 times). A further PBS wash was followed by blocking non-specific binding sites using 5% BSA/PBS for 1 h at room temperature. The sections were incubated with HRP-conjugated secondary antibody for 1 h at room temperature after being incubated with the primary antibody at 4 °C throughout the night, followed by blocking with goat antiserum for 1 h at room temperature. DAB chromogenic reaction was performed, and observation/imaging was carried out using a light microscope. Specific antibodies and their dilution ratios are provided in Supplementary Table 1.

### 2.10 Gut microbiome, non-targeted metabolomics, and transcriptomics analyses

Integrated multi-omics analyses, including microbiome profiling, non-targeted metabolomics, targeted bile acid metabolomics, and transcriptomics sequencing, were performed on murine colon tissues and colonic contents to elucidate the regulatory mechanisms of EUPs in FMT-induced colitis (*n* = 6/group). Briefly, 0.2 g of distal colonic content was weighed for the extraction of metabolites and microbial DNA. All omics assays were conducted by Wuhan Maiwei Metabolic Biotechnology Co., Ltd., with raw data processed and visualized on the proprietary Maiwei Cloud bioinformatics platform. All statistical assessments were carried out using R software (v4.2.1), and statistical significance was determined at the *p* < 0.05 level.

#### 2.10.1 Sequencing-based gut microbiome profiling

To analyze microbial community structure and species profiles, we performed high-throughput sequencing on the conserved V3–V4 hypervariable segment within the 16S rRNA gene, following the method described previously (Zhao T. et al., 2024; Zhao Y. et al., 2024). Before launching amplification reactions, genomic DNA was retrieved from colonic content samples via extraction, with concentration quantified via a NanoDrop spectrophotometer and integrity checked through 1% agarose gel electrophoresis. The V3–V4 region was amplified with primers 338F/806R (TaKaRa Ex Taq^™^ HS) in a 25 μL PCR system, using cycling conditions (95 °C 5 min; 72 °C 10 min; 30 cycles: 95 °C/55 °C/72 °C for 30 s/30 s/45 s); Amplicon quality was validated via restriction enzyme digestion to confirm amplification specificity and reliability, followed by quantification using an Agilent Bioanalyzer 2100. We conducted high-throughput sequencing with an Illumina NovaSeq 6000 sequencer (California, United States) to generate 2 × 300 bp paired-end reads for downstream taxonomic and functional annotation. OTU (Operational Taxonomic Unit) clustering was conducted at 97% sequence similarity with UPARSE (v7.1), species annotation was performed against the Silva database (v138), α-diversity indices (Shannon, Simpson, Chao1) were calculated using QIIME2 (v2022.2) with statistical differences assessed by one-way ANOVA, and β-diversity was assessed via Bray–Curtis dissimilarity-based PCoA with significance determined through PERMANOVA.

#### 2.10.2 Non-targeted metabolomics sequencing

We carried out intestinal metabolic profiling via ultra-performance liquid chromatography-tandem mass spectrometry as described previously (Yan et al., 2024). Metabolites from 0.2 g colonic contents were extracted via 1 mL methanol: water (4:1, v/v), 30 min sonication (4 °C), and 15 min centrifugation (13,000 × g, 4 °C). UPLC separation used an ACQUITY BEH C18 column at 40 °C, with a solution running at a flow rate of 0.3 mL/min, and gradient elution. An analyzer was employed to examine samples in electrospray ionization (ESI). The metabolite identification was achieved by matching MS/MS fragmentation patterns and *m*/*z* values to the KEGG metabolite database, achieving an overall diagnostic accuracy of 80%. To ensure data reproducibility, three biological replicates were used for analyzing each sample, and supplementary technical replicates were used for critical chromatographic runs to reduce experimental variability, with an intra-batch CV that fell below the 15% cutoff value.

#### 2.10.3 Bile acids sequencing

Targeted bile acid profiling (GC, as described previously) included homogenizing 0.15–0.2 g fecal pellets of each mouse were mixed with 500 μL of ultrapure water and vortexed for thorough blending (2 min). The samples were amalgamated with pure ether and 100 μL 5 mol/L H₂SO₄ solution, subjected to centrifugation (3,000 × g for 10 min, 4 °C), and then vigorously agitated. The upper ether phase was concentrated to 0.2 mL via nitrogen evaporation, and the collected samples were moved to vials for gas chromatography analysis. Bile acid standards (cholic acid, chenodeoxycholic acid, etc.) from Sigma-Aldrich (United States) were used to establish calibration curves with *R*^2^ > 0.99, while quantification was conducted via the external standard method, and recovery rates were calculated to range from 85 to105% with a CV <10%.

#### 2.10.4 Transcriptomics sequencing (RNA-seq)

0.2 g of colon tissue samples were subjected to RNA sequencing. TRIzol reagent was extracted for total RNA extraction, followed by purification with the RNeasy Mini Kit. We evaluated RNA integrity by means of an Agilent 2100 Bioanalyzer and RNA 6000 Nano Kit (Agilent Technologies, 5067-1511, United States), with a strict inclusion standard set at an RNA integrity number (RIN) of no less than 7.0 and implemented for samples to be used in cDNA library preparation. Expression levels were quantified on an Illumina platform, and gene expression levels were normalized and quantified as the FPKM using HTSeq (v0.14.1) for the subsequent differential expression procedures. Differential expression analysis via DESeq2 (v1.34.0) was implemented using the criteria (|log₂ (fold change) | >1, FDR <0.05, VIP score >2.0), and functional annotation of DEGs relied on GO and KEGG pathway enrichment analyses via clusterProfiler (v4.2.2).

### 2.11 Statistical analysis procedure

GraphPad Prism 7 software was used to conduct statistical tests, with all experimental data presented as mean ± SD (standard deviation). Statistical tests were run using GraphPad Prism 7 software, and comparisons between groups were completed with an unpaired Student’s *t*-test to compute relevant *p*-values. Statistical significance is denoted by asterisks (^*^p < 0.05, ^**^p < 0.01, ^***^p < 0.001, and ^NS^*p* > 0.05). Sample size estimation was performed using G*Power 3.1 software. By setting effect size to *d* = 0.8, significance level *α* = 0.05, and statistical power = 0.8, the sample size per group was ultimately determined to be *n* = 6–10 mice per group. Additionally, normality of data and homogeneity of variances were evaluated via the Kolmogorov–Smirnov test and Levene’s test, respectively.

## 3 Results

### 3.1 Impact of EUPs on East Frisian sheep FMT-induced colitis in mice models

Sheep are commonly used as a ruminant model for studying gastrointestinal microbiota and intestinal inflammatory diseases. In recent years, studies have reported that the core gut microbiota functions (metabolism of Bacillota and Bacteroides) in ruminants such as sheep are highly conserved in mammals compared to those in humans and mice. Although there are differences in composition, the core functions such as short-chain fatty acid synthesis and mucosal immune regulation are consistent (Mizrahi et al., 2021). Previous studies have established a DSS-induced enteritis model in sheep. Typical clinical manifestations of colitis (diarrhea, weight loss, elevated DAI), characteristic histopathological lesions (mucosal erosion, inflammatory cell infiltration, crypt damage), and a UC-like intestinal microbial dysbiosis pattern were all observed in DSS-treated sheep. We further performed fecal microbiota transplantation (FMT) in mice with feces from control and DSS-treated sheep. FMT from DSS-induced sheep recapitulated a colitis phenotype and intestinal dysbiosis like human UC in recipient mice (Yan et al., 2024). Consistent outcomes were observed following FMT in cattle (Zhao et al., 2022a, 2022b; Zhao X. et al., 2022). FMT is an attractive strategy for correcting the dysbiosis of the microbiota in patients with irritable bowel syndrome mainly characterized by diarrhea (Singh et al., 2022).

**Figure 1:**
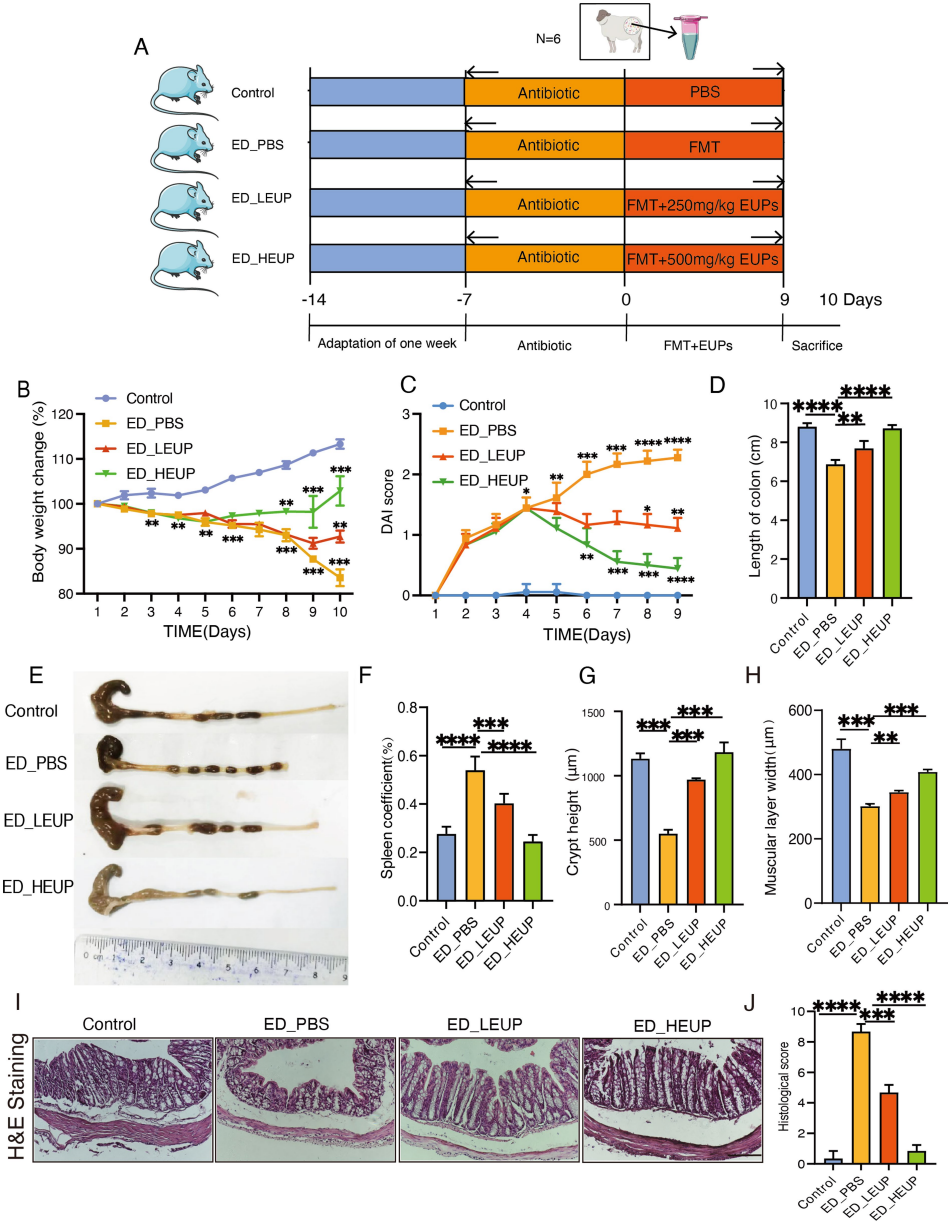
EUPs alleviated mice subjected to East Frisian sheep FMT-induced colitis. **(A)** Experiment design schedule. **(B)** Body weight change curve. **(C)** DAI score in mice. **(D,E)** Representative colon pictures and colon length in each group. **(F)** Spleen coefficient in each group. **(G–J)** Crypt height, muscular layer width, H&E staining, and histological activity index (HAI) in the colon of mice. Scale bar = 100 μm. All the above experiments were repeated three times independently, and the data were expressed as “mean ± standard deviation (SD).” (^*^p < 0.05, ^**^p < 0.01, ^***^p < 0.001, and ^****^p < 0.0001) (*n* = 10 samples/group).

To further validate these findings, we investigated the therapeutic effects of two doses of EUPs in mice with colitis induced by FMT. As depicted in Figure 1A, the effect of EUPs on mice subjected to East Frisian sheep FMT-induced colitis was evaluated. For deeper exploration of EUPs’ effect on intestinal inflammation, research results indicate that the ED-PBS group (ED-PBS group was given DSS treatment East Frisian sheep fecal liquid) significantly altered mice subjected to East Frisian sheep FMT-induced colitis phenotype, including reduced body weight (by 83% ± 0.41% g) (Figure 1B), increased DAI scores (by 2.26 ± 0.10) (Figure 1C) (Supplementary Figure 1A) and shorter colon length (by 6.5 ± 0.10 cm) (Figures 1D,E). In contrast, the ED-LEUP (ED-LEUP group was administered DSS exposure East Frisian sheep the fecal microbial suspension + low concentration EUPs) and ED-HEUP groups (ED-HEUP group was administered DSS exposure East Frisian sheep the fecal microbial suspension + high concentration EUPs) treatment significantly mitigated body weight loss (by 91% ± 0.11% g and 102% ± 0.13% g, respectively) (Figure 1B), reduced DAI scores (by 1.16 and 0.5, respectively) (Figure 1C) and improved colon length (by 7.5 cm and 8.4 cm, respectively) compared to the ED-PBS group (Figures 1D,E). Interestingly, compared with the ED-PBS group, ED-LEUP and ED-HEUP significantly reduced the spleen coefficient (Figure 1F). H&E staining showed colonic mucosal injury, colonic crypt absence, intestinal gland deformation, inflammatory cell infiltration, and a high histological injury score in the ED-PBS group. ED-LEUP and ED-HEUP groups significantly reduced the overall colonic histological score, decreased mucosal injury, and reduced inflammatory cell infiltration (Figures 1G–J). Serum biochemical analysis showed no significant differences across the groups (Supplementary Figures 1B–I). These outcomes suggest that the EUPs is capable of effectively attenuating colitis symptoms through improving weight loss, colon length parameters, and DAI among FMT mice without noticeable side effects.

### 3.2 EUPs strengthen the intestinal barrier function in mice subjected to East Frisian sheep FMT-induced colitis

**Figure 2:**
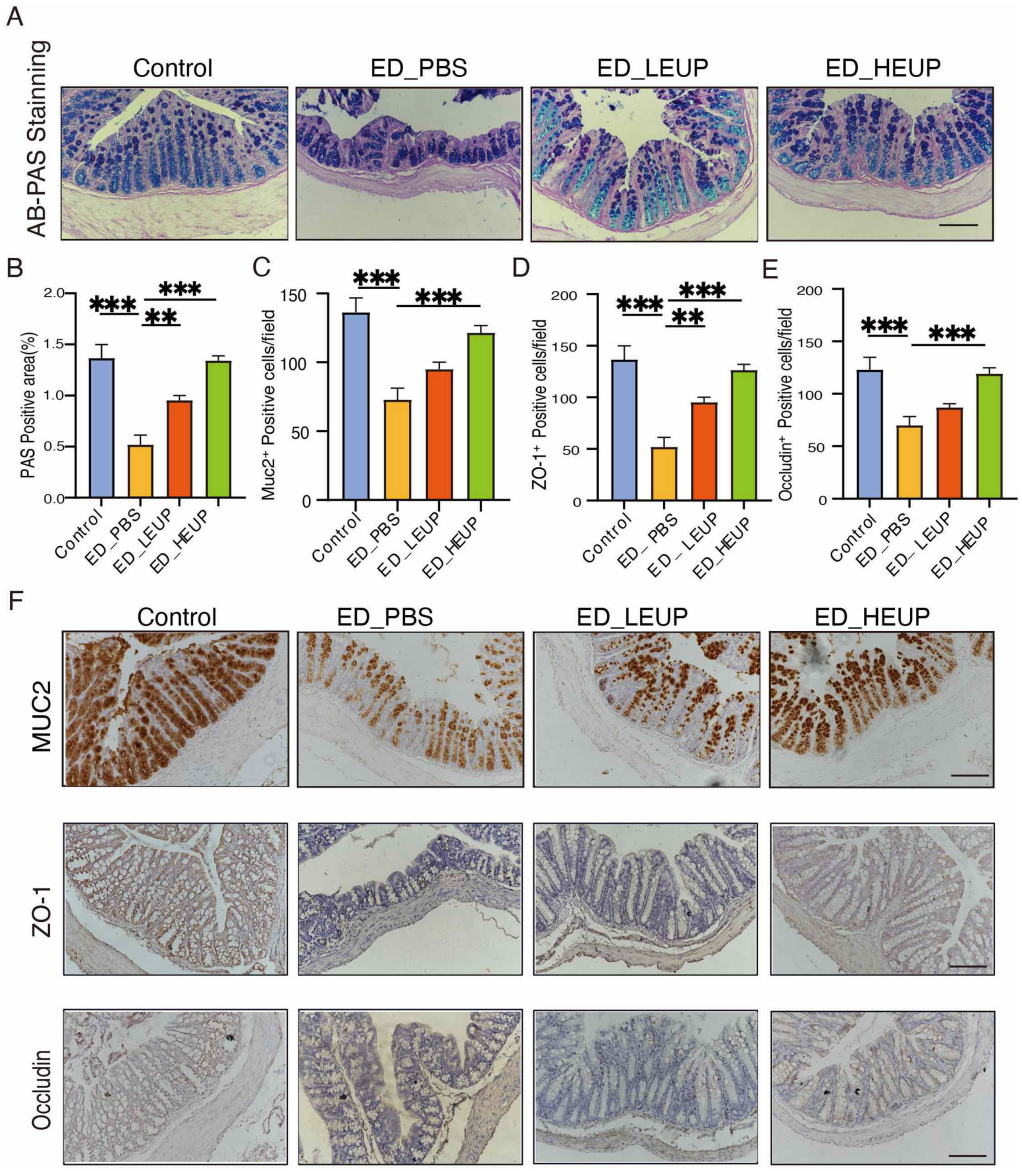
EUPs improved the intestinal barrier integrity in mice subjected to East Frisian sheep FMT-induced colitis. **(A,B)** AB-PAS staining and quantification of positive cells per field in the colonic tissue. Scale bar = 100 μm. **(C–F)** Immunohistochemical analysis for the distribution of MUC2, Occludin, and ZO-1 with quantitative analysis in colon tissues among the four groups. Scale bar = 100 μm. All the above experiments were repeated three times independently, and the data were expressed as “mean ± standard deviation (SD).” (^*^p < 0.05, ^**^p < 0.01, ^***^p < 0.001, and ^****^p < 0.0001) (*n* = 6 samples/group).

Intestinal immune homeostasis imbalance and intestinal barrier impairment constitute important pathological features of colitis (Abbas et al., 2025). As an important intestinal mucosal protein component, MUC2 serves as a physical barrier to prevent microorganisms in the intestinal epithelium (Sheikh et al., 2022). Via AB-PAS staining, the ED-PBS group exhibited decreased colonic mucus amount and goblet cell numbers, whereas the administration of two doses EUPs elevated the mucin-positive cell amount and goblet cell count to different degrees (Figures 2A,B). To better evaluate how EUPs protect against enteritis, levels of ZO-1 (TJP1), mucin2 (MUC-2), and Occludin (OCLN) protein expression were quantified by IHC. The outcomes suggest that expression of Occludin, MUC-2, and ZO-1 markedly decreased in the ED-PBS group. In contrast, the EUPs treatment notably increased these expression levels. The ED-HEUP group showed a significant inhibitory effect on the decrease in colonic MUC-2, ZO-1, and Occludin (Figures 2C–F). These experimental results demonstrated that EUPs can restore colonic barrier integrity in mice subjected to East Frisian sheep FMT-induced colitis.

### 3.3 EUPs alleviate the colon flora disorder in mice subjected to East Frisian sheep FMT-induced colitis

**Figure 3:**
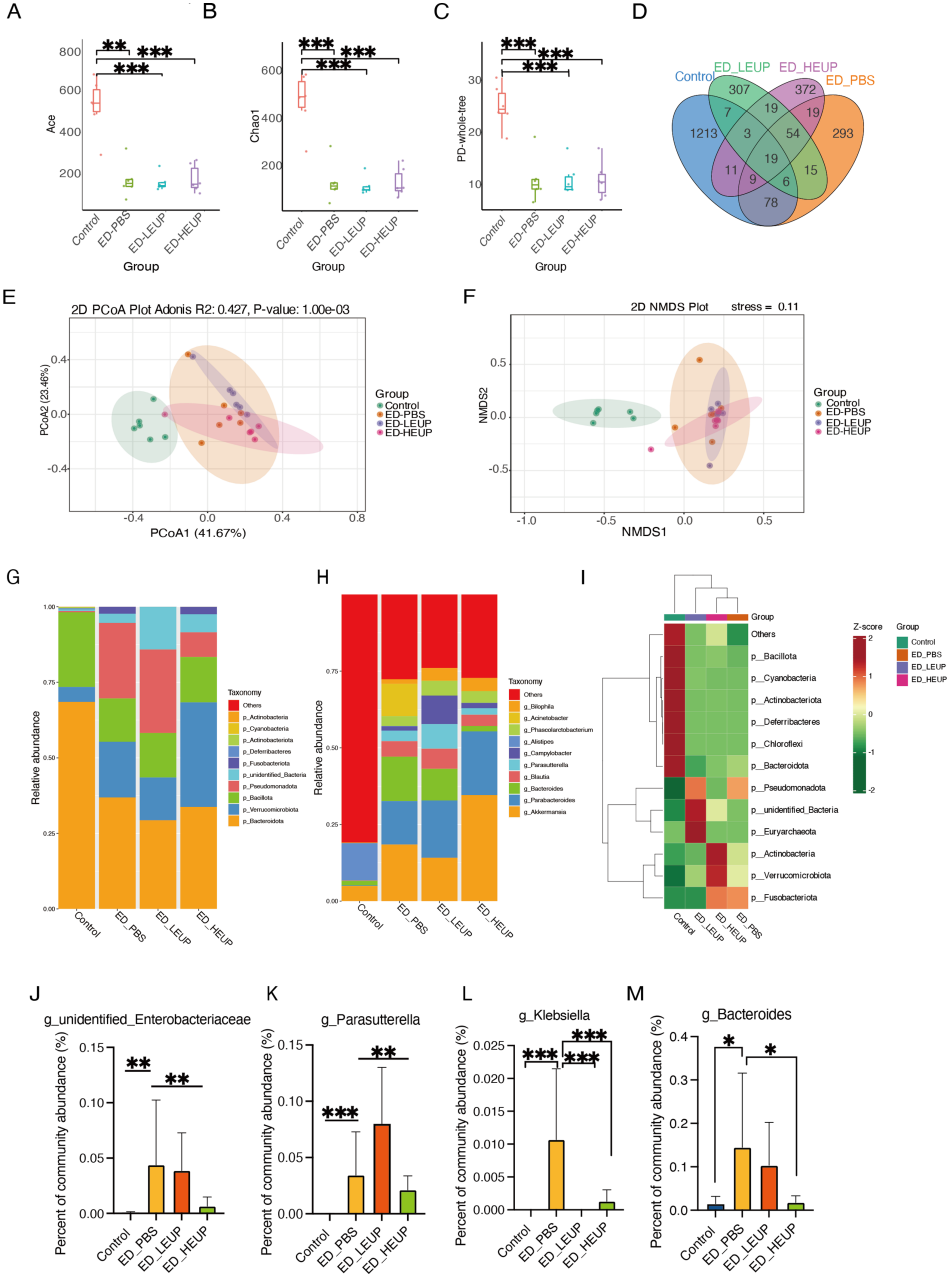
EUPs alleviated the intestinal flora disorder in mice subjected to East Frisian sheep FMT-induced colitis. **(A–C)** Microbial α-diversity analysis. **(D)** Venn diagram of colonic microbial analysis among the four groups. **(E,F)** The PCoA plot showed the β-diversity of colonic microorganisms. **(G,H)** The top 10 microbiota taxa at the phylum and genus levels. **(I)** Heatmap of the gut microbiota at the phylum level. The color gradient represents the *Z*-score standardized value of the relative expression level (red: high expression, blue: low expression). **(J–M)** The abundance of key bacteria of *g_unidentified_Enterobacteriaceae*, *g_Parasutterella*, *g_Klebsiella*, and *g_Bacteroides* at the genus level. All the above experiments were repeated three times independently, and the data were expressed as “mean ± standard deviation (SD).” (^*^p < 0.05, ^**^p < 0.01, and ^***^p < 0.001) (*n* = 6 samples/group).

Intestinal microbiota is critical for both the pathological progression and repair of intestinal injury, and microbiota functional imbalance or maintenance of its homeostasis is closely associated with the injury (Ijssennagger et al., 2015). To assess whether EUPs regulate the microbiota composition of mice subjected to East Frisian sheep FMT-induced colitis, we conducted microbiome sequencing. The α-diversity analysis revealed that the ED-PBS group exhibited significantly lower levels of the ACE, Chao1, and PD-whole-tree indices, while EUPs treatment significantly increased these indices. Moreover, the ED-HEUP group has a more significant effect on these indices in mice subjected to East Frisian sheep FMT-induced colitis (Figures 3A–C). Microbial community composition was examined for each group, revealing 1,213 group-specific microorganisms in the Control cohort, 293 unique microbes in the ED-PBS cohort, 307 in the ED-LEUP cohort, and 372 in the ED-HEUP cohort, with 19 common microorganisms present across all four groups (Figure 3D). We carried out β-diversity analysis to evaluate differences in intestinal flora composition among various groups. There was a significant discrepancy in colon microbiota composition when comparing the Control with the ED-PBS groups. Notably, following EUPs intervention, the overall intestinal microbiota in the two groups exhibited a compact clustering pattern with good sample reproducibility, and the composition profile of colon microbiota in ED-HEUP was comparable to the Control group (Figures 3E,F). Across the four groups, relative abundances of colon-resident microbiota changed markedly at both phylum and genus taxonomic ranks (Figures 3G,H). From the phylum-level perspective, within the ED-PBS cohort, p_Pseudomonadota abundance exhibited a significant increase, whereas p_Bacillota relative abundances displayed a notable drop. Interestingly, the ED-HEUP group exhibited an increase in the relative abundance of p_Bacillota, as well as a reduction in the relative abundance of p_Pseudomonadota, indicating that two doses of EUPs active ingredients exert distinct effects on colon microbiota composition (Figure 3I). At the genus taxonomic rank, harmful microbial taxa such as *g_unidentified_Enterobacteriaceae*, *g_Parasutterella*, *g_Klebsiella*, and *g_Bacteroides* exhibited significantly higher relative abundances in the ED-PBS cohort compared with the Control cohort. In contrast, the EUPs treatment caused a significant decline in *g_Parasutterella*, *g_Klebsiella*, *g_unidentified_Enterobacteriaceae*, and *g_Bacteroides* (Figures 3J–M).

**Figure 4:**
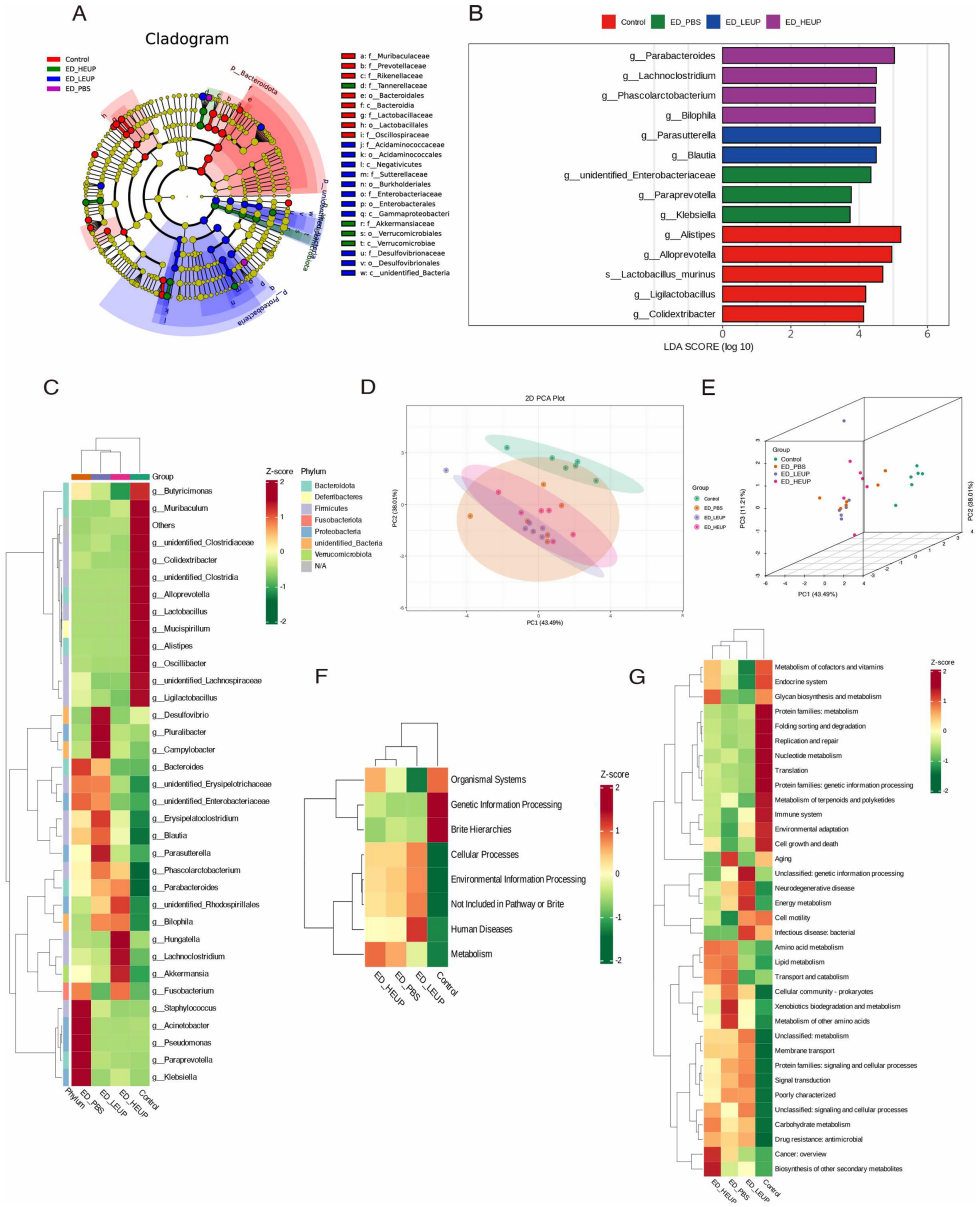
Differences in microbial abundance and functional annotation from PICRUSt2 analysis on the associations in gut microbiota. **(A,B)** Cladogram plot of differentially abundant species and linear discriminant analysis (LDA) effect size (LEfSe) of the colonic microorganisms among the four groups. **(C)** Heatmap of the gut microbiota at the genus level (red: high expression, blue: low expression). **(D,E)** Demonstration of PICRUSt2 (Phylogenetic Investigation of Communities by Reconstruction of Unobserved States) functional annotation PCA results based on ASV at primary functional classification. **(F,G)** PICRUSt2 predicted the gut microorganisms’ function at levels 1 and 2.

LEfSe (linear discriminant analysis effect size) analysis demonstrated that the ED-PBS group underwent a marked increase in the relative abundance of *s_Parabacteroides_goldsteinii*, *g_Klebsiella*, and *g_unidentified_Enterobacteriaceae* relative to the Control cohort. The ED-HEUP group, additionally, showed a notable increase in the relative abundance *g_Parabacteroides, f_Akkermansiaceae*, *o_Verrucomicrobiae*, and *g_Lachnoclostridium* (Figures 4A,B). The heat map also showed the same result (Figure 4C). Microbial function analysis revealed that colon microbial functions differed between the ED-PBS and ED-HEUP groups, while the colon microbial functions between the Control and ED-HEUP groups were similar (Figures 4D,E). Functional characterization of the complete gut microbiota, using the KEGG database, revealed that intestinal microbiota exhibited a broad range of physiological functions. When compared with the Control group, the ED-PBS group displayed statistically significant enrichment in the metabolic pathways of cofactors and vitamins, the endocrine system, and glycan biosynthesis metabolism. In terms of metabolic pathway profiles, the ED-HEUP group presented significant enrichment in the endocrine system, cofactors and vitamins, and glycan biosynthesis metabolism pathways. The ED-LEUP group presented a marked decrease in lipid metabolism, transport, and catabolism, and amino acid metabolism pathways compared with the ED-PBS group (Figures 4F,G).

### 3.4 EUPs regulate colon metabolites in mice subjected to East Frisian sheep FMT-induced colitis

**Figure 5:**
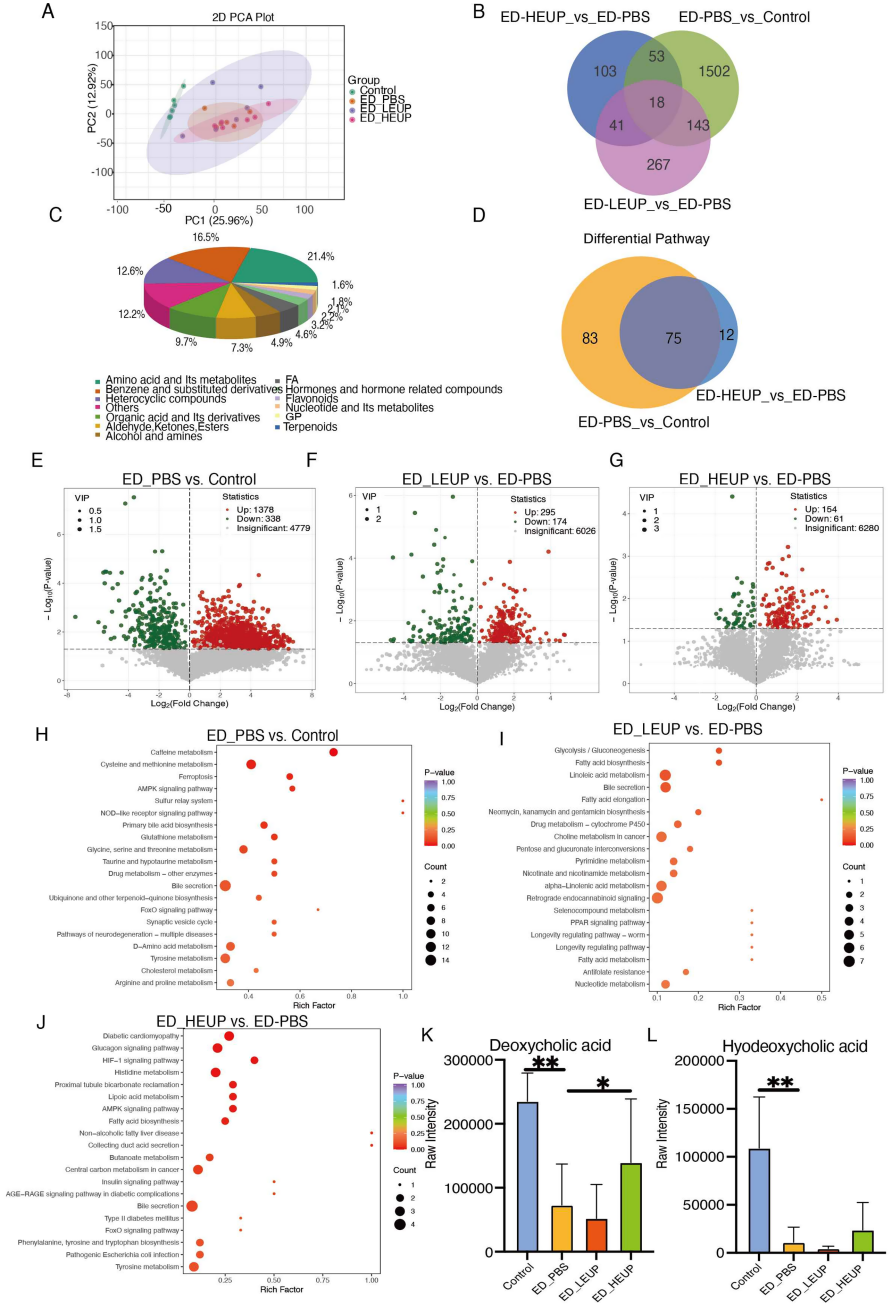
EUPs regulated intestinal metabolites in mice subjected to East Frisian sheep FMT-induced colitis. **(A)** PCoA plots of the colonic metabolites among the four groups of mice. **(B)** Venn diagram of differential colonic metabolites analysis among the four groups of mice. **(C)** Pie chart of metabolites classification. Each color in the pie chart represents the differential classification, and the area represents the relative proportion of the metabolites. **(D)** Venn diagram of differential colonic metabolite pathways enrichment analysis. **(E–G)** Volcanic diagram of colon metabolites among the four groups of mice (FDR <0.05 and |log2FoldChange| >1, Benjamini–Hochberg FDR method). **(H–J)** The KEGG pathway analysis of differentially expressed metabolites among the four groups of mice. **(K,L)** Relative concentrations of deoxycholic acid and hyodeoxycholic acid in the colon. The data were expressed as “mean ± standard deviation (SD).” (^*^p < 0.05, ^**^p < 0.01, and ^***^p < 0.001) (*n* = 6 samples/group).

To investigate EUPs’ treatment potential mechanisms among the four groups, we carried out non-targeted metabolomics analysis on colon contents. The level of intestinal metabolites was shown to have changed significantly via PCA (Figure 5A). The Venn diagram-based overlap analysis further confirmed the consistency of these differential metabolite profiles, yielding congruent results (Figure 5B). The classification of these 7,737 metabolites revealed that 21.4, 9.7, and 4.9% were organic acid and its derivatives amino acid and its metabolites, and alcohol and amines, respectively (Figure 5C). Differential metabolic pathways also demonstrated the same result (Figure 5D). Differential metabolite set analysis indicated 1,378 upregulated and 338 downregulated differentially abundant metabolites were detected in the ED-PBS group when compared with the Control group (Figure 5E). When assessed against the ED-PBS group, 295 upregulated and 174 downregulated metabolites were present in the ED-LEUP group (Figure 5F), and 154 upregulated and 61 downregulated metabolites were identified in the ED-HEUP group (Figure 5G). For exploring functions of these altered metabolites, results showed that differential metabolites were primarily enriched in cysteine and methionine metabolism, ferroptosis, primary bile acid biosynthesis, and the AMPK signaling pathway in the ED-PBS group versus the Control group (Figure 5H). When contrasted with the ED-PBS group, the ED-LEUP group was significantly enriched in the glucagon signaling pathway, fatty acid biosynthesis, lipoic acid metabolism, the bile secretion, and collecting duct acid secretion pathways (Figure 5I). Moreover, compared to the ED-PBS group, the ED-HEUP group was mainly enriched in the bile acid metabolism pathway (Figure 5J). The same changes have also been shown in colon metabolites, specifically including deoxycholic acid and hyodeoxycholic acid, which are key bile acids (Figures 5K,L).

### 3.5 EUPs improve the production of bile acid in mice subjected to East Frisian sheep FMT-induced colitis

**Figure 6:**
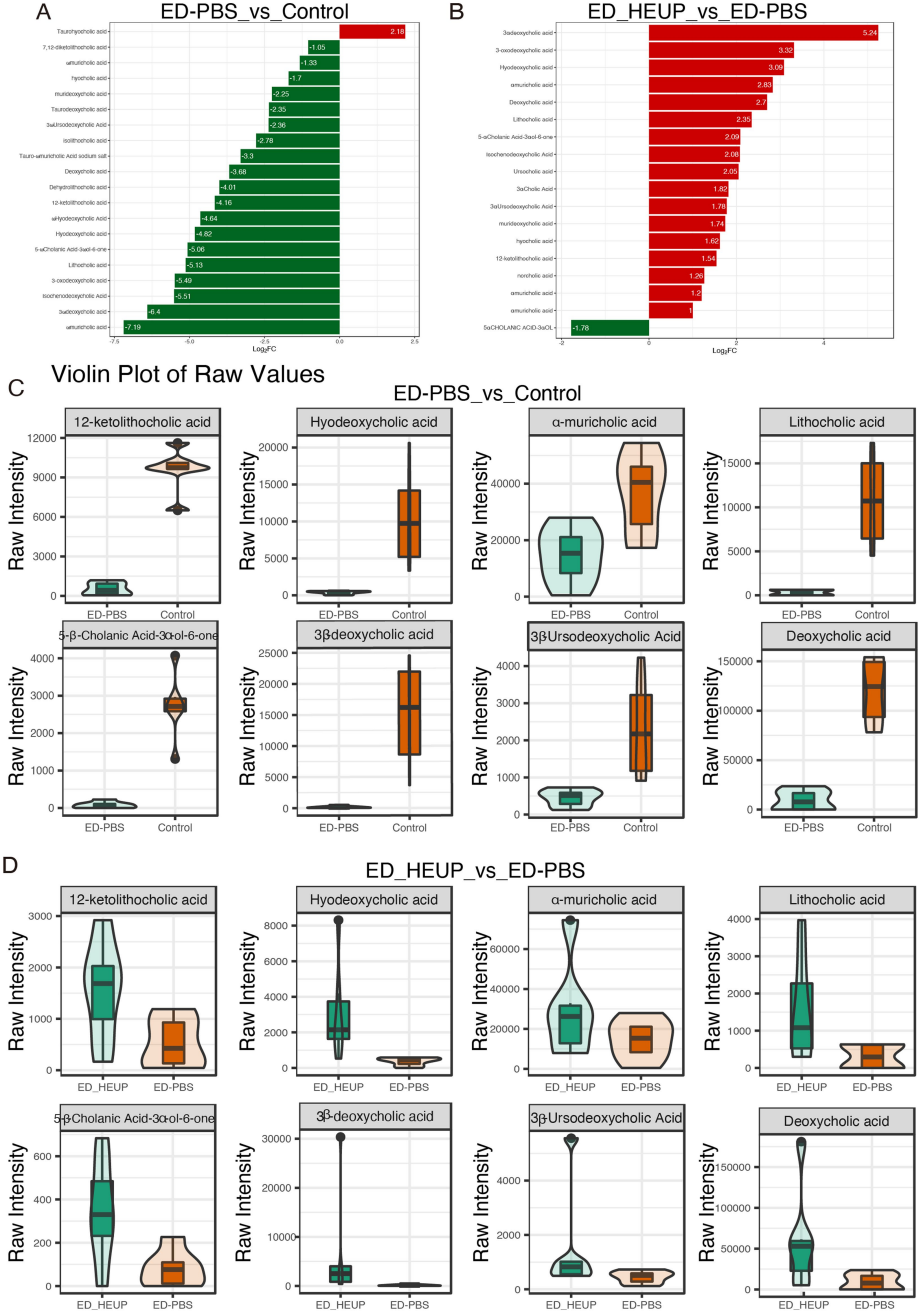
EUPs improved the bile acid profile in mice subjected to East Frisian sheep FMT-induced colitis. **(A,B)** A volcano plot of the significantly differentially regulated metabolites among the three groups of mice. **(C,D)** Quantification of 12-ketolithocholic acid, hyodeoxycholic acid, α-muricholic acid, lithocholic acid, 5-β-cholanic acid 3-α-ol-6-one, 3β-deoxycholic acid, 3β-ursodeoxycholic acid, and deoxycholic acid concentrations in the colonic contents of the Control groups, ED-PBS group, and ED-HEUP groups (^*^p < 0.05, ^**^p < 0.01, ^***^p < 0.001, and ^****^p < 0.0001) (*n* = 6 samples/group).

Bile acids serve a role in suppressing the excessive proliferation of intestinal bacteria (Zhao T. et al., 2024; Zhao Y. et al., 2024). To investigate how EUPs influence bile acid production, we conducted targeted metabolomics analysis on colon contents. The levels of 19 BAs were notably downregulated in the ED-PBS group (Figure 6A). In contrast, 17 BAs displayed a notable elevation in their levels within the ED-HEUP group (Figure 6B). For example, the levels of several specific BAs were significantly decreased, such as hyodeoxycholic acid, α-muricholic acid (α-MCA), 5-β-cholanic acid 3-α-ol-6-one (5β-CA-3α-ol-6-one), 3β-deoxycholic acid (3β-DCA), 12-ketolithocholic acid (12-KLCA), lithocholic acid (LCA), 3β-ursodeoxycholic acid (3β-UDCA), and deoxycholic acid in the ED-PBS group, however, ED-HEUP treatment reversed this trend (Figures 6C,D). These results demonstrated that EUPs treatment can effectively regulate bile acid production, including deoxycholic acid and hyodeoxycholic acid, thereby alleviating colitis.

### 3.6 The mechanism of EUPs treatment in mice subjected to East Frisian sheep FMT-induced colitis was revealed via RNA-seq analysis

**Figure 7:**
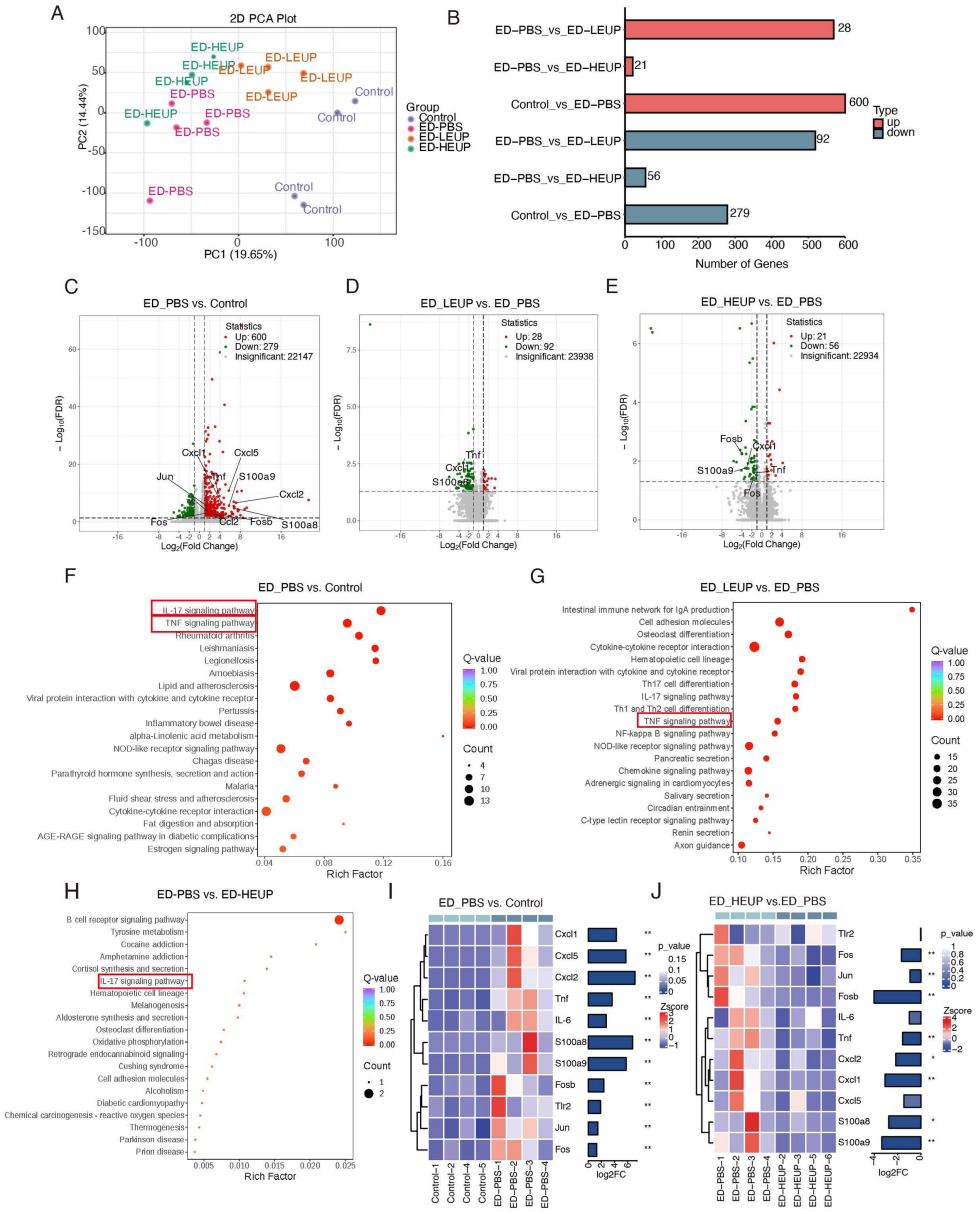
RNA-seq analysis revealed the mechanism of EUPs treatment in mice subjected to East Frisian sheep FMT-induced colitis. **(A)** PCA plots of colonic distinct gene expression in the Control, ED-PBS, ED-LEUP, and ED-HEUP groups. **(B)** Statistical chart of differential colonic genes among the four groups of mice. **(C–E)** A volcano plot of the significantly differentially regulated genes among the four groups of mice. **(F–H)** The KEGG pathway analysis of differentially expressed genes among the four groups of mice. **(I,J)** Heatmap of the IL-17 signaling pathway in the colon between the ED-PBS group versus the Control, and the ED-HEUP versus the ED-PBS (*n* = 6 samples/group).

To further explore how EUPs exert a protective effect against colitis, colon tissue specimens underwent RNA-seq profiling. PCA plot revealed substantial variations in gene expression patterns between the Control and ED-PBS cohorts, whereas the ED-LEUP and ED-HEUP groups clustered closely (indicating minimal differences in their gene expression profiles). Additionally, clear segregation of ED-LEUP and ED-HEUP groups from the ED-PBS group was apparent, which reflected pronounced disparities in their gene expression profiles (Figure 7A). The ED-PBS group had 879 significantly different genes relative to the Control group, including 279 downregulated and 600 upregulated ones. In comparison with the ED-PBS group, 120 genes were found to exhibit marked expression discrepancies in the ED-LEUP group (92 downregulated, 28 upregulated), and the ED-HEUP group had 77 significantly different genes (21 upregulated, 56 downregulated) (Figures 7B–E).

To further characterize the functional roles of differentially expressed genes, a KEGG enrichment analysis was carried out on the genes exhibiting differential abundance. Research findings revealed that the ED-PBS group was predominantly enriched in the interleukin-17, TNF, and NOD-like receptor signaling pathways, while EUPs mitigated this alteration (Figure 7F). In comparison with the ED-PBS group, the ED-LEUP group was found to have substantial enrichment in the TNF, cytokine-cytokine receptor interaction, and IL-17 signaling pathways (Figure 7G). By contrast, primary enrichment in B cell receptor, IL-17, and cell adhesion molecules pathways was observed in the ED-HEUP group. Interestingly, enrichment of the TNF signaling pathway was not detected here (Figure 7H). Excitingly, heat map analysis of signaling pathways further revealed that all critical genes linked to the IL-17 pathway, including *IL-6*, *CXCL1*, *CXCL2*, *CXCL5*, *TNF*, *S100A8*, *S100A9*, *JUN*, and *FOS*, were all downregulated in the ED-HEUP group (Figures 7I,J).

We carried out Spearman’s rank correlation tests to clarify the connections between microorganisms, metabolites, and host genetic changes. These results revealed that potentially beneficial bacteria, including *Alloprevotella*, *Alistipes*, *Ligilactobacillus*, and *Colidexribacter*, were positively correlated with beneficial metabolites, particularly bile acids (12-ketolithocholic acid, hyodeoxycholic acid, α-muricholic acid, deoxycholic acid). Conversely, *g_Parasutterella*, *g_unidentified_Enterobacteriaceae*, and *g_Klebsiella* exhibited a negative correlation with these metabolites (hyodeoxycholic acid, deoxycholic acid) (Supplementary Figure 2A). Furthermore, *g_Parasutterella*, *g_unidentified_Enterobacteriaceae*, and *g_Klebsiella* exhibited a negative correlation with genes correlated to the IL-17 pathway, particularly *CXCL1*, *CXCL2*, *IL-6*, *TNF*, *JUN*, and *FOS* (Supplementary Figure 2B). The metabolites of 12-ketolithocholic acid, hyodeoxycholic acid, α-muricholic acid, and deoxycholic acid also displayed an inverse correlation with related to the IL-17 signaling pathway (Supplementary Figure 2C).

## 4 Discussion

UC impacts millions of individuals worldwide, and its incidence continues to escalate globally (Fan et al., 2024). However, the fundamental mechanism by which pathogenic microorganisms or symbiotic microorganisms are involved in this context remains unknown. To mimic specific pathological features of UC, we established FMT-induced colitis by using fecal samples from DSS-treated sheep as the experimental model. Furthermore, current data regarding the effects of the EUPs on ulcerative colitis remain scarce, particularly with respect to their impacts on gene expression patterns, profiles of microbial metabolites, and the composition of gut microbiota. To elucidate the underlying mechanism of EUPs in ameliorating colitis, a suite of experimental and analytical approaches were adopted, including the use of germ-free mice, 16S rRNA sequencing, untargeted metabolomics, targeted metabolomics, and colon transcriptomics analysis.

Initially, as prior research has indicated that colitis development is independent of gender, we chose C57BL/6J male mice as the experimental model to minimize potential confounding effects associated with sex differences (Sang et al., 2014). Growing research evidence points to gut microorganisms fulfilling a pivotal function in initiating gut inflammatory pathogenesis (Wang et al., 2020). Consistently, intestinal inflammation seen in the ED-PBS group has also been observed in various other experimental enteritis models (Wu et al., 2025). The ED-PBS group induces mucosal inflammation and intestinal barrier damage, raising endotoxemia levels and systemic inflammation, and thus leads to the onset of enteritis (Menezes-Garcia et al., 2020). However, the adverse effects of antibiotics on beneficial flora and persistent concerns surrounding food safety present formidable obstacles to the successful treatment of colitis, necessitating alternative therapeutic approaches. Previous studies have shown that *Cistanche tubulosa* polysaccharide (*C. tubulosa*) can alleviate intestinal damage (Liu et al., 2025), while *Astragalus* polysaccharides have been identified as capable of ameliorating ulcerative colitis (Zhang et al., 2025). This study showed that EUPs exhibit a potent anti-inflammatory effect by decreasing colon histological scores, improving intestinal crypts, and mucosal integrity, consistent with prior research (Abbasi-Kenarsari et al., 2020). Consistent with our research, epithelial cells are interconnected to tight junction proteins that can inhibit the invasion of harmful substances, including Occludin and ZO-1 (Han et al., 2024).

Disruption of gut microbiota homeostasis is a key pathological feature in enteritis. In this study, EUPs supplementation markedly decreased the harmful bacteria at the genus level, including *g_Klebsiella*, and *g_unidentified_Enterobacteriaceae*. In patients suffering from colitis, the prevalence of Pseudomonadota in their colonic mucosa is elevated (Abad et al., 2024). The potential mechanism via which Bacteroidetes alleviates colitis may involve the utilization of sulfatases to remove sulfate groups from mucins, thereby promoting mucin degradation and maintaining the intestinal mucus barrier (Luis et al., 2021). Moreover, the heightened abundance of Pseudomonadota in colitis may induce epithelial cells to secrete pro-inflammatory mediators and chemokine ligand 20, thereby facilitating a mucosal immune response and neutrophil recruitment, which can further intensify intestinal inflammation (Scher et al., 2013). Consistent with this study, EUPs treatment partially restored a healthy gut microbial profile in the colitis mice.

Intestinal flora dysbiosis is generally regarded as a main cause of metabolic disorders. Firstly, this conclusion was demonstrated in the microbial function prediction of this study. Serving as a symbiotic component of the intestinal microbiota, it contributes substantially to maintaining mucosal barrier function and modulating immune responses by generating beneficial metabolites (e.g., BAs) (Rooks and Garrett, 2016). Metabolomic PCA analysis revealed good intra-group clustering for both the ED_HEUP and ED-PBS groups, yet no significant separation was detected between these two groups, a result that may reflect the characteristic regulatory pattern of EUPs. Similar trends have been documented in research focused on natural product-driven gut metabolism regulation: polysaccharide treatment preserves intestinal homeostasis not through sweeping alterations to the metabolomic landscape, but via targeted modulation of critical anti-inflammatory metabolite levels, including bile acids and short-chain fatty acids (Peng et al., 2019). To further examine the roles of metabolites, a KEGG analysis indicates that EUPs treatment significantly altered metabolic pathways (fatty acid biosynthesis, collecting duct acid secretion, and bile secretion). In line with prior findings, our results showed that the ED-HEUP group significantly increased in bile acid metabolism, with key representatives (deoxycholic acid, hyodeoxycholic acid). Bile acids, which are essential beneficial metabolites, can modulate intestinal inflammation, barrier integrity, and cellular proliferation (Dong et al., 2021). Numerous research projects have shown that the decline of certain bile acids (e.g., deoxycholic acid) disrupts gut ecological imbalance and provokes the initiation of colitis (Connors et al., 2020). However, a high-fat diet has been reported to elevate intestinal DCA concentrations by nearly 10-fold, and chronic exposure to high colonic DCA levels in mice can trigger marked intestinal inflammation that recapitulates key features of human inflammatory bowel disease (Li et al., 2023). Therefore, we conducted targeted metabolomics quantification in the intestinal contents of mice. The results showed that, compared with previous studies, the concentrations of DCA and HDCA at the physiological level were relatively low, and the low concentrations could significantly enhance the viability of IEC-6 cells and tight junction proteins (Traub et al., 2008). As a primary bile acid, hyodeoxycholic acid can be converted into secondary bile acids (e.g., DCA) by intestinal flora and modulates intestinal immunity through the FXR-TGR5 axis (Chiang, 2017). For bile acid-tolerant bacteria (e.g., Bacteroidetes), an increase in these microbes leads to an elevation in the level of bile acids, indicating that such microorganisms may interfere with bile acid metabolism (Quraishi et al., 2020).

Microbial dysbiosis disrupts the physical and immunological barriers of the gut (Zhao et al., 2022a, 2022b; Zhao X. et al., 2022). For further exploring potential associations between the intestinal flora, host immune response, and intestinal barrier function, we conducted colonic transcriptomics studies. One of the key findings is that the ED-HEUP group underwent a significant reduction in the IL-17 signaling pathway. We noted that EUPs administration significantly downregulated these genes (*JUN*, *FOS*, *IL-6*, *IL-17*), known as IL-17 signaling modulators and hub genes, and most of these genes play critical roles in mediating inflammatory responses. The IL-17 cascades play critical roles in inflammatory diseases, particularly intestinal inflammation, and modulating the IL-17 pathway offers a promising therapeutic avenue for treating intestinal inflammation (Mitsuyama et al., 1991). Differing from *Astragalus* polysaccharides, which exert anti-colitis effects via enriching SCFA-producing gut bacteria and regulating Treg/Th17 equilibrium (Zhang et al., 2025). Gut microbiota-mediated activation of the PI3K/Akt axis underlies the ability of the novel *Atractylodes macrocephala* fructan to alleviate ulcerative colitis (Wu et al., 2026). Distinct from other polysaccharides, EUPs exhibit a unique regulatory pattern by targeting key microbial strains (e.g., *g_unidentified_Enterobacteriaceae* and *g_Klebsiella*) and preferentially modulating the production of secondary bile acids (DCA, HDCA), which distinguishes their unique regulatory mode to modulate the IL-17 pathway. Of note, a robust positive association is observed between the beneficial intestinal flora in the host’s colon and the colonic metabolites of bile acids (deoxycholic acid and hyodeoxycholic acid). Low-dose EUPs (250 mg/kg) still enriched the TNF pathway, likely due to an inadequate dose failing to fully reverse microbiota imbalance. High-dose EUPs (500 mg/kg), by contrast, notably reduced pathogenic bacteria, including *g_Klebsiella* and diminished TNF-α release. Based on the mechanisms of targeted binding efficiency, inhibition of pro-inflammatory signaling pathways, and enhanced regulation of the intestinal microbiota and intestinal barrier repair, 500 mg/kg EUPs may be more effective in alleviating colitis. However, it has some limitations: We do not know whether the potential beneficial changes in intestinal microbiota and metabolites following the administration of EUPs are directly regulated by these microbes and their metabolites or are attributable to secondary immune effects. Although EUPs modulated the activity of the IL-17 pathway, the precise mechanism underlying their regulatory effects remains unclear. Furthermore, further studies will employ a more complete set of dose gradients (e.g., low, medium, high, and ultra-high groups) to better define the optimal therapeutic dosage of EUPs, thereby offering valuable guidance for future in-depth research. Finally, the existing results from animal experiments have not completely demonstrated that they possess therapeutic efficacy in humans and East Frisian sheep. Future research should further validate these findings through the following approaches: Both *in vitro* intestinal organoid assays and *in vivo* validation experiments in sheep further confirmed EUPs’ therapeutic activity. Bile acid receptor antagonists were used to modulate FXR/TGR5, aiming to observe whether the IL-17 pathway could be restored. Fecal microbiota from the ED-HEUP group mice or key microbial taxa was transplanted into DSS-induced colitis mice, confirming that EUPs-modulated microbiota serves as the core mediating factor.

## 5 Conclusion

Our study demonstrates that the administration of EUPs can effectively alleviate colitis in mice subjected to East Frisian sheep FMT-induced colitis, achieved by attenuating key colitis symptoms, restoring intestinal barrier integrity, altering the colon microbiota composition, increasing the level of bile acids, and inhibiting the IL-17 signaling pathway.

## Data Availability

The datasets supporting the conclusions of this article are publicly available. The raw sequencing data were deposited in the NCBI Sequence Read Archive (SRA) under accession numbers PRJNA1087596 (RNA-seq raw data) and PRJNA1087159 (microbiota raw sequencing data); The metabolomics data were deposited in the MetaboLights database under accession number MTBLS9748 (Untargeted metabolomics data).
