# Supplementary material for: Eucommia polysaccharides alleviate experimental colitis by reshaping colonic microbiota composition, metabolites, and modulating the IL-17 signaling pathway
Source: Front Microbiol. 2026 Mar 25;17:1769429. doi: 10.3389/fmicb.2026.1769429 (PMC13057415; doi:10.3389/fmicb.2026.1769429)
Supplement: Supplementary file 1 [file Supplementary_File_1.docx]

Supplementary Material

# Supplementary Figures and Tables

## Supplementary Figure1.


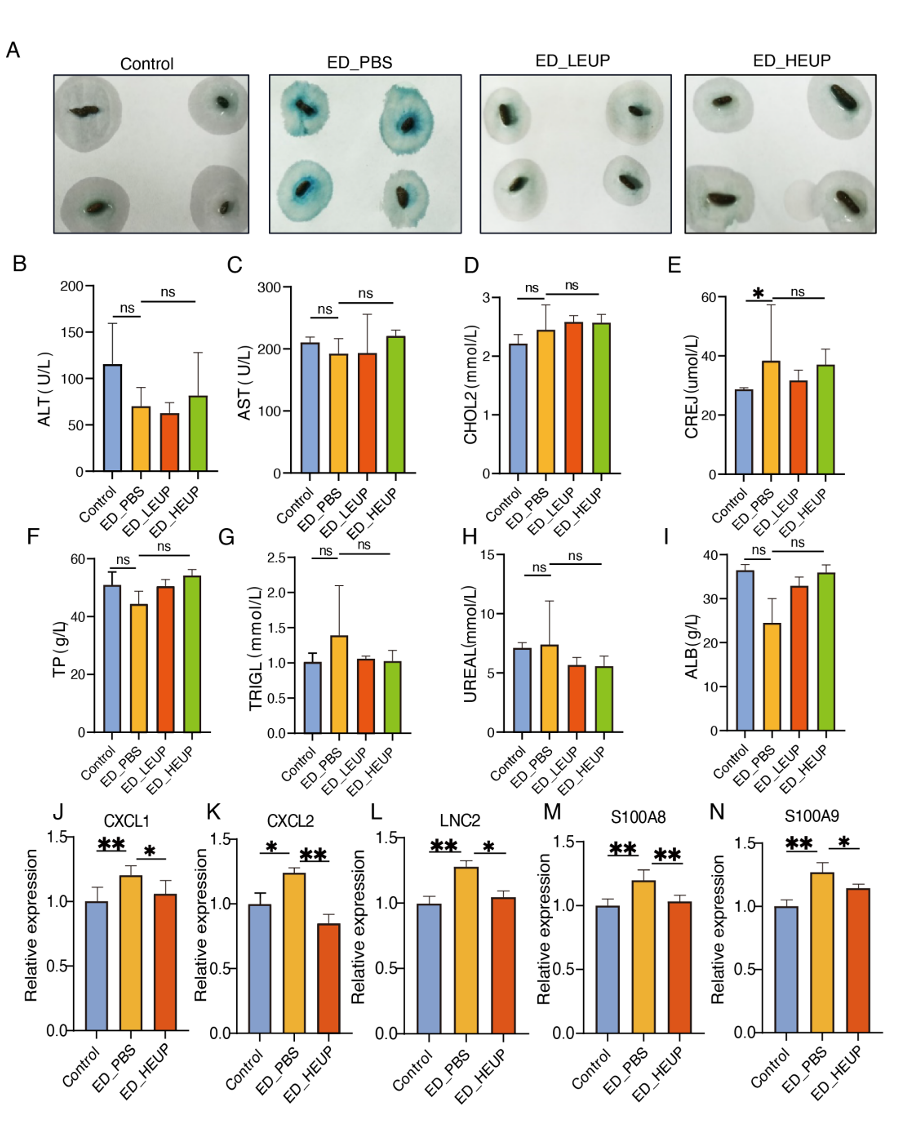


**Supplementary Figure 1.** Occult blood in the stool of mice and serum biochemistry. (A) Changes in occult blood in the stool of mice on the fifth day. (B-I) Serum biochemistry was evaluated based on ALT, AST, CHOL2, CREJ, TP, TRIGL, UREAL and ALB levels among the four groups of mice. All the above experiments were repeated three times independently and the data were expressed as “Mean ± standard deviation (SD)”. **P* < 0.05, ***P* < 0.01, *** *P* < 0.001.


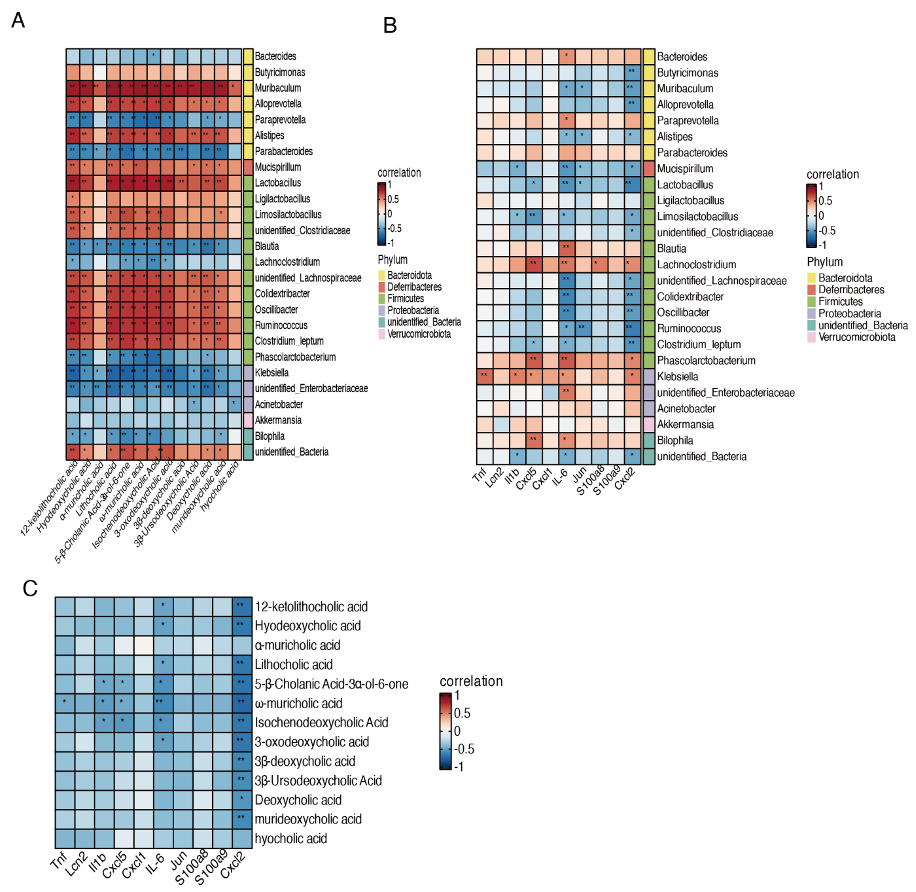
**1.2 Supplementary Figure2**

**Supplementary Figure 2.** Spearman’s correlation analysis. Correlation between differentially abundant genera and (A) bile acids. (B) Genes. (C) Correlations between genes and bile acids.

**1.3 Additional Files 1**

**Additional Files 1. Primary antibody for immunostaining**

| Name | Dilution ratio |
| --- | --- |
| Anti-ZO-1 (proteintech，21773-1-AP) | 1:500/1:1000 |
| Anti-Occuldin (proteintech，13050-1-AP) | 1:500/1:1000 |
| Anti-MUC2 (Servicebio，GB11344) | 1:500/1:1000 |
